# Serotonin Signaling in *Schistosoma mansoni*: A Serotonin–Activated G Protein-Coupled Receptor Controls Parasite Movement

**DOI:** 10.1371/journal.ppat.1003878

**Published:** 2014-01-16

**Authors:** Nicholas Patocka, Nidhi Sharma, Mohammed Rashid, Paula Ribeiro

**Affiliations:** Institute of Parasitology, McGill University, Macdonald Campus, Ste. Anne de Bellevue, Quebec, Canada; University of Pennsylvania, United States of America

## Abstract

Serotonin is an important neuroactive substance in all the parasitic helminths. In *Schistosoma mansoni*, serotonin is strongly myoexcitatory; it potentiates contraction of the body wall muscles and stimulates motor activity. This is considered to be a critical mechanism of motor control in the parasite, but the mode of action of serotonin is poorly understood. Here we provide the first molecular evidence of a functional serotonin receptor (Sm5HTR) in *S. mansoni*. The schistosome receptor belongs to the G protein-coupled receptor (GPCR) superfamily and is distantly related to serotonergic type 7 (5HT7) receptors from other species. Functional expression studies in transfected HEK 293 cells showed that Sm5HTR is a specific serotonin receptor and it signals through an increase in intracellular cAMP, consistent with a 5HT7 signaling mechanism. Immunolocalization studies with a specific anti-Sm5HTR antibody revealed that the receptor is abundantly distributed in the worm's nervous system, including the cerebral ganglia and main nerve cords of the central nervous system and the peripheral innervation of the body wall muscles and tegument. RNA interference (RNAi) was performed both in schistosomulae and adult worms to test whether the receptor is required for parasite motility. The RNAi-suppressed adults and larvae were markedly hypoactive compared to the corresponding controls and they were also resistant to exogenous serotonin treatment. These results show that Sm5HTR is at least one of the receptors responsible for the motor effects of serotonin in *S. mansoni*. The fact that Sm5HTR is expressed in nerve tissue further suggests that serotonin stimulates movement via this receptor by modulating neuronal output to the musculature. Together, the evidence identifies Sm5HTR as an important neuronal protein and a key component of the motor control apparatus in *S. mansoni*.

## Author Summary

The bloodfluke *Schistosoma mansoni* causes human schistosomiasis, a debilitating disease that afflicts over 200 million people worldwide. There is no vaccine for schistosomiasis, and chemotherapy relies heavily on a single drug, praziquantel. With only one drug available, the prospect of drug resistance is a serious concern, particularly when praziquantel usage is on the rise due to mass treatment programs in many parts of the world. There is a pressing need to identify new drug targets and to develop new chemotherapeutics for schistosomiasis. The focus of this research is on the nervous system of *S. mansoni*. Many pesticides and antiparasitic drugs act by interacting with neuronal proteins and therefore the nervous system holds great promise for drug discovery. Here we describe a new protein that is present in the nervous system of *S. mansoni* and regulates movement of the worm. The protein was further identified as a specific receptor for serotonin, an important neurotransmitter and a known modulator of motility in schistosomes. This work shows that the serotonin receptor of *S. mansoni* is required for proper motor control and therefore is a potential target for chemotherapeutic intervention.

## Introduction

Schistosomes belong to the phylum platyhelminths and reside in the intravascular system of the host. They affect more than 200 million people worldwide with a death toll of over 250,000 per year [1]–[3]. As many as five species of schistosomes are known to infect humans, the most common species being *Schistosoma mansoni, S. hematobium, and S. japonicum*. The disease is caused by the paired adult worms releasing large numbers of eggs that become trapped in surrounding tissues, notably the liver and spleen. There is no vaccine for schistosomiasis and treatment relies heavily on the use of a single drug, praziquantel. Concerns over the prospect of praziquantel resistance have renewed interest in the search for new chemotherapeutics and alternative control strategies [see 4, 5].

The nervous system of schistosomes has strong potential for drug targetting, particularly those elements of the nervous system that control muscle function and movement. Schistosomes undergo lengthy and complex migrations in the bloodstream of the mammalian host, a behavior that is tightly linked to their development and continued propagation. The coordination of muscle function, movement and sensory inputs required for successful navigation during this migration are all controlled by the nervous system of the worm. Besides movement, schistosomes use their neuromuscular systems to control the muscles of the suckers, which allow the worm to attach to the host, the muscle lining of the viscera, including reproductive, digestive and excretory tracts, and also the tight coupling of males and females. Disruption of any one of these activities through pharmacological intervention could interfere with the normal life cycle and potentially cause elimination of the parasite from the host. Many of the anthelmintics used for treatment of nematode infections work by disrupting the worm's neuromuscular system, including such mainstays as ivermectin, levamisole and monepantel [6], [7].

Like other flatworms, schistosomes have a well-developed central nervous system (CNS) consisting of bilobed cerebral ganglia and several pairs of longitudinal nerve cords connected by transverse commissures. The CNS, in turn, links to an extensive network of peripheral nerve fibers and neuronal plexuses that supply virtually every region of the body, notably the body wall muscles responsible for movement [8]. At the molecular level, the schistosome nervous system employs many of the same signaling molecules and receptors that occur in other organisms, including neuropeptides and small-molecule (classical) neurotransmitters [9]. Among the classical transmitters, serotonin (5-hydroxytryptamine: 5HT) has received the most attention because of its wide distribution in the schistosome nervous system and its well-documented effects on worm motility. Serotonin is a derivative of the amino acid, tryptophan, and a common neuroactive substance in many animal phyla. Evidence dating back to the 1960s has shown that serotonin is strongly myoexcitatory in all the flatworms and is present both in the CNS and peripheral nervous system (PNS) of the worm, including the lateral nerve fibers and plexuses of the PNS that supply the body wall muscles [10]–[20]. In schistosomes, serotonin was shown to stimulate motility when applied exogenously onto intact adult worms or sporocysts in culture [14]–[16], it potentiated muscle contraction in preparations of body wall muscle strips and isolated muscle fibers [18], [19] and it also stimulated glucose uptake and carbohydrate metabolism [21], thus making more energy available for muscle contraction. Two molecular components of the serotonergic system have been identified in *S. mansoni*, including the rate-limiting enzyme in serotonin biosynthesis [22] and a plasma membrane serotonin transporter [23], [24], which is expressed in serotonin neurons and mediates neuronal reuptake of the transmitter [25]. There is also indirect, largely pharmacological evidence that serotonin stimulates adenylate cyclase and increases cAMP in crude flatworm extracts [26], [27] but this has yet to be verified by molecular methods. Serotonin receptors have not been cloned or characterized at the molecular level in any of the parasitic flatworms.

In other species, serotonin exerts its effects by interacting with cell-surface receptors from two different protein superfamilies, either G protein-coupled receptors (GPCRs) or ligand-gated ion channels. The vast majority of known vertebrate and invertebrate serotonin receptors are rhodopsin-like (Family A) GPCRs and share a common topology characterized by seven transmembrane (TM) domains, the N-terminus being extracellular and the C-terminus intracellular. In vertebrates there are seven major types of serotonin receptors (named 5HT1-5HT7), of which six are GPCRs [28]. This classification is based on structure, signaling mechanism and also pharmacological properties. Invertebrates have fewer receptor types [29] and their classification is more challenging because the sequences are divergent and the invertebrate receptors do not always recognize classical (i.e. mammalian) serotonergic agonists and antagonists. To date only three major types of serotonergic GPCRs have been identified in invertebrates. These are distantly related to mammalian 5HT1, 5HT2 and 5HT7 receptors, which represent the three canonical mechanisms of serotonergic signaling. 5HT1 is negatively coupled to adenylate cyclase and inhibits cAMP production, whereas 5HT7 stimulates cAMP and 5HT2 signals through the inositol phospholipid (IP_3_) and Ca^2+^ signaling pathway [28], [29]. Molluscs, insects and nematodes have all three types of serotonergic GPCRs and the mechanisms of signaling are conserved when the receptors are expressed heterologously in mammalian cells [29]. Among the flatworms there is molecular evidence for multiple types of 5HT receptors in free-living planarians [30], [31] but the mechanisms of signaling have not been elucidated and it is unknown if these receptors are conserved in the parasites.

Here we show for the first time that a GPCR cloned from *S. mansoni* is selectively activated by serotonin and stimulates production of cAMP in transfected mammalian cells. The *S. mansoni* serotonin receptor (Sm5HTR) is structurally related to previously described planarian receptors [30], [31] and is a distant homologue of mammalian 5HT7. We further demonstrate that Sm5HTR is abundantly expressed throughout the nervous system of the worm and it is required for proper control of motor activity, as determined by RNA interference (RNAi). The results identify Sm5HTR as the major mechanism by which serotonin stimulates movement in schistosomes.

## Materials and Methods

### Parasites

*Biomphalaria glabrata* snails infected with a Puerto Rican strain of *S. mansoni* were obtained from the Biomedical Research Institute (Bethesda, MD, USA). Cercarial shedding was performed 6 weeks post-infection, as previously described [32]. Cercariae were mechanically transformed to obtain larval schistosomulae [32] and cultured under previously optimized conditions [33]. To obtain adult worms, female CD1 mice were infected with approximately 150–200 cercariae/mouse and worms were harvested 6–8 weeks post infection by portal perfusion [32]. Animal procedures were reviewed and approved by the Facility Animal Care Committee of McGill University (Protocol No. 3346) and were conducted in accordance with the guidelines of the Canadian Council on Animal Care

### Cloning of Sm5HTR and expression in HEK293 cells

A BLAST analysis of the *S. mansoni* genome database [34], [35] revealed a predicted rhodopsin-like GPCR (Smp_126730) that shared significant sequence homology with known serotonin receptors from other species. The sequence was thus termed Sm5HTR and cloned from reverse-transcribed *S. mansoni* cDNA. Total RNA was isolated from adult male and female pairs, using TRIZOL (Invitrogen), and reverse-transcribed with MMLV reverse transcriptase (Invitrogen) and Oligo-(dt)_12–18_ primer, according to standard procedures. The resulting cDNA was used as a template to amplify the coding sequence of Sm5HTR with sequence-specific primers. The primer sequences targeting the beginning and end of the predicted coding sequence are as follows: sense primer (5′-ATGACAATCTCACAATTGG); antisense (5′-TCATCTTTCATCCGTTTGACC). The resulting product was cloned into p-GEM-T-easy vector (Promega) and the sequence was confirmed for three separate clones. For expression in HEK 293 cells, the full length Sm5HTR cDNA was modified by PCR to introduce a Kozak sequence for optimal translation efficiency in mammalian cells, followed by a FLAG fusion tag (DYKDDDDK) at the receptor's N-terminal end. The modified cDNA was cloned into the expression vector pCI-neo between the XhoI and NotI sites and verified by sequencing. HEK 293 cells were seeded in six-well plates and transfected with 3 µg of either empty plasmid or pCI-neo-Sm5HTR, using Fugene6 transfecting agent (Roche) and the manufacturer's protocols. Approximately 48 h–72 h post-transfection, the cells were tested for Sm5HTR expression by *in situ* immunofluorescence, using an anti-FLAG antibody (Sigma), followed by FITC conjugated anti-mouse IgG secondary antibody, as described previously [23].

### cAMP assays

HEK 293 cells were transfected with pCI-Neo-Sm5HTR or empty plasmid (mock), as above. Cells were harvested 48 h post-transfection, seeded in 96-well plates at a density of 100,000 cells/well and cultured overnight at 37°C. The next day they were used for cAMP assays with the CatchPoint™Cyclic-AMP Fluorescent Assay kit (Molecular Devices), following the manufacturer's recommendations. Briefly, cells were washed once in Kreb's-Ringer Bicarbonate buffer supplemented with glucose and were subsequently treated with 0.75 mM 3-isobutyl-l-methylxanthine (IBMX) prepared in the same buffer for 10 minutes at room temperature. Cells were washed again in buffer and treated with 20 µM forskolin and test substances at 10^−4^ M (unless otherwise noted) for 10 minutes at 37°C. Antagonists were tested in the presence of 10^−4^ M serotonin. Following incubation, cells were washed, and lysed. Aliquots of the resulting cell lysates were tested for cAMP by a competitive immunoassay using rabbit anti-cAMP antibody followed by Horseradish Peroxidase (HRP)-cAMP conjugate and a fluorescent substrate, as per the kit protocol. Fluorescence was measured in a FlexStation microplate reader set at λ_em_ = 485 nm and λ_ex_ = 554 nm. Quantitative amounts were calculated from a standard curve of known amounts of cAMP (provided with kit). Each assay was performed in duplicates or triplicates and was replicated through at least three independent experiments.

### Antibody production and western blot analyses

A polyclonal anti-Sm5HTR antibody was produced in rabbits against two synthetic peptides (Twenty-first Century Biochemicals, MA, USA). Peptide 1 (CKAREYDKRLNSYSS) is located in the third intracellular loop, while peptide 2 (CKRQSIVISSPYTRND) is located in the C-terminal tail of the Sm5HTR protein. The peptides were conjugated to ovalbumin as a carrier and tested for specificity using BLAST analysis against the Schistosome Genome Database [34], [35] and the general protein database at NCBI (National Center for Biotechnology Information). Recognition of the peptides was tested by ELISA and the antiserum was found to be of high titer. The IgG fraction specific to Sm5HTR was subsequently purified using peptide-conjugated beads (Sigma), according to standard procedures, and the eluted fractions were tested again by ELISA. For western blot analyses, we extracted membrane proteins from adult *S. mansoni* (males and females) using the ProteoExtract Native Membrane Protein Extraction kit (EMD Millipore Bioscience), as described in the kit protocol. Aliquots of membrane proteins were resolved on a 4–12% gradient Tris-Glycine precast gel (Invitrogen) and transferred to polyvinylidene fluoride (PVDF) membranes (Millipore). Blots were probed using affinity purified anti-Sm5HTR antibody (1∶1000 dilution) followed by HRP-labeled goat anti-rabbit IgG antibody (1∶25,000) (Calbiochem). Negative controls were performed with an excess of combined peptide antigen (0.25 mg/ml of each peptide). For comparative analyses of Sm5HTR protein expression in RNAi experiments, western blots were probed first with affinity-purified anti-Sm5HTR antibody, as above, then stripped and re-probed with an antibody against a different *S. mansoni* membrane protein (SmSERT [25]) as a loading control.

### Confocal microscopy

Freshly recovered adult worms and *in vitro* transformed schistosomulae (3- and 8-day old) were fixed in 4% paraformaldehyde for 4 hours at 4°C. Following fixation, samples were washed three times in PBS, treated with 1% SDS (prepared in PBS) for 20 min at room temperature, washed three times in antibody diluent (1× PBS pH 7.4, 0.5% bovine serum albumin, 0.5% triton X-100, 0.1% sodium azide) and then washed once more in 100 mM Glycine (prepared in PBS) to minimize background fluorescence. Samples were placed in blocking buffer (antibody diluent containing 1% BSA) for 2 hours at room temperature and then overnight at 4°C. Affinity-purified anti-Sm5HTR antibody was added in blocking buffer (1∶100) and the incubation continued for 3 days at 4°C with end/over end rotation. Samples were washed three times with antibody diluent and incubated for 2 additional days with fluorescein isothiocyanate (FITC) - labeled donkey anti rabbit antibody (1∶1000) dilution. In dual labeling experiments, the worms were treated with anti-serotonin monoclonal antibody (Abcam) (1∶200) and peptide-purified anti-Sm5HTR, followed by incubation with secondary antibodies conjugated to FITC and Alexa Fluor 594 (1∶1000). In other experiments animals were labeled with 4′,6-diamidino-2-phenylindole, dihydrochloride (DAPI) (1∶750) to stain cell nuclei or TRITC-labeled phalloidin (200 µg/ml) to visualize the musculature. In both cases the stains were added during the last day of incubation with the secondary antibody. Animals were further washed with antibody diluent 5 times (10 min each at room temperature), then mounted with anti-quenching mounting media (Sigma) and visualized using either a BioRad Radiance 2100 confocal scanning microscope and the Lasersharp 2000 software package (BioRad, Canada) or a Zeiss LSM710 confocal microscope equipped with Zen 210 software (Carl Zeiss Inc., Canada). For dual labeling experiments, the confocal images were acquired using a sequential acquisition mode and two separate lasers. Filter sets were optimized to eliminate fluorescence spectral overlap and bleed-through artifacts. Controls used include anti-Sm5HTR preadsorbed with an excess of pooled peptide antigens (0.25 mg/ml of each peptide) and secondary antibody alone.

### RNA interference (RNAi)

RNAi was performed both in larvae and adult worms. For RNAi in larvae, newly transformed schistosomulae were transfected with a synthetic siRNA (Qiagen) targeting the third intracellular loop (IL3) of Sm5HTR (positions 863–883 of the coding sequence; 5′-GAGAAGCTCATGGAAGAAGAT-3′) or an irrelevant (scrambled) siRNA (Ambion) as a negative control. The transfection was performed as described previously [36], using siPORT lipid transfection reagent (Ambion) and 50 nM gene-specific or control siRNA. A mock-transfected control (transfection reagent only) and an untransfected control lacking both siRNA and transfection reagent were also prepared. The test and control cultures were supplemented with 10% fetal bovine serum (FBS) the day after transfection and left for an additional 7 days (8 days in total) before analysis. For RNAi in adult worms, freshly collected males and females were electroporated with siRNA, as described [37]. We used the same custom designed Sm5HTR siRNA described above and, as a negative control, we used a pool of endonuclease-digested siRNAs against an irrelevant sequence (mCherry). The pooled siRNAs were prepared as described previously [36]. Briefly, we amplified a 203 bp fragment of the common reporter, mCherry by PCR, using primers that introduced flanking T7 promoters at both ends. The PCR product was *in vitro* transcribed with T7 RNA polymerase, according to standard protocols, and the resulting mCherry dsRNA was digested with Dicer (RNAse III) (Ambion) and purified, according to the manufacturer's recommendations. For electroporation, unpaired males and females (approximately 5 of each) were placed in a 4 mm cuvette in 100 µl RPMI media containing equal amounts (5 µg) of gene-specific or control siRNAs and a single square-wave 20 ms impulse of 125 V was applied at room temperature. Following electroporation, the worms were transferred to a 12-well culture plate containing 1 ml of complete RPMI medium per well (RPMI supplemented with 10 mM Hepes, 2 mM glutamate, 5% BSA, 100 U/ml penicillin and 100 mg/ml streptomycin) and cultured for about 24 hr at 37°C prior to analysis. In preliminary experiments, the adult worms were examined at different time points between 16 hr and up to 5 days post-electroporation and 24 hr was found to produce optimal motor phenotypes under the conditions tested.

### Quantitative PCR (qPCR)

RNAi was routinely monitored by RT- qPCR analysis both in larvae and adult worms. Total RNA was isolated using a commercial extraction kit (Qiagen), quantified with a Nanodrop ND1000 spectrophotometer (Wilmington,USA) and approximately 100–500 ng of total RNA was used for the RT reaction. The RT was performed according to standard protocols in a 20 µl reaction using 200 U M-MLV reverse transcriptase (Invitrogen) and oligodT primer. Real-time qPCR was subsequently performed using the Platinum SYBR Green qPCR SuperMix-UDG kit (Invitrogen) in 25 µl reactions. Primers were designed to amplify a 256 bp region in the Sm5HTR coding sequence (forward 5′- GAACACCACAACGTATGGC- 3′ and reverse 5′- CCTGCTGTCATTTTTGACT- 3′) or a 201 bp fragment of *S. mansoni* α-tubulin (Accession# S79195; forward 5′-TCGTGGTGATGTTGTCCCCAAG-3′ and reverse 5′-TCGGCTATTGCGGTTGTATTAC-3′). The qPCR was performed in a Rotor-Gene RG3000 instrument (Corbbet Research) with the following cycling conditions: 50°C/2 min, 95°C/2 min, followed by 40 cycles of 95°C/30 s, 56.7°C/30 s, and 72°C for 30 sec. Primer validation curves were generated for each primer set to estimate amplification efficiencies of the target Sm5HTR and housekeeping gene (α-tubulin). Expression data were normalized relative to the housekeeping gene and the relative differences in expression were finally calculated by the Pfaffl's method [38]. All RT-qPCR data are derived from a minimum of three independent experiments, each in triplicates.

### Motility assays

Motility assays were performed using a compound microscope (Nikon, SMZ1500) equipped with a digital video camera (QICAM FAST 1394, mono 12 bit, Qimaging) and SimplePCI version 5.2 (Compix Inc.) for image acquisition. Animals were videoed at a rate of 3 frames/s either for 1 min (schistosomulae) or 2 min (adult worms) and the resulting images were imported into ImageJ (version 1.41, NIH, USA) for further analysis. Schistosomulae exhibit a type of movement in culture that is dominated by repeated shortening and elongation of the body. We quantified this movement by measuring the frequency of length changes (shortening and elongation) per min of observation, as described previously [25], [39]. For measurements of adult worm motility, we developed a relatively simple imaging assay based on ImageJ. First the videos were processed to minimize effects of variable illumination intensity, using the ‘Stack deflicker’ function of the ‘wrMTrck’ plugin in ImageJ. Next, the background was subtracted and the resulting images were converted to binary format by using a thresholding algorithm available in ImageJ. Frames were inspected manually through this process to insure that worms were accurately represented as binary objects. Adult schistosomes exhibit complex motor behaviors in culture that involve irregular thrashing and whip-like movements. To quantify this type of movement we used the “Subtract” algorithm of “Image Calculator” in Image J. The software measures the difference in pixels between two consecutive frames by subtracting frame “n” from frame “n+1” through the entire video. The subtracted pixels represent a change in the position of the body relative to the previous frame and we used this measurement to obtain an estimate of worm movement. A schematic of how the images were processed is shown in Fig. S1. In a typical experiment we recorded video from 5 males and 5 females per well and then analyzed worms individually to obtain an average number of subtracted pixels per frame over the two minutes of observation. Motility was calculated as the average number of subtracted pixels/frame relative to the total number of pixels in the binary object to account for variation in worm size. Experiments were repeated three times in duplicates, each well with at least 5 males and 5 females to obtain average motility values. To measure the effect of serotonin and other related substances on schistosome (adult or larval) motility the animals were treated with test substance for 10 min at room temperature prior to recording of movement. Agonists and antagonists were added at 100 µM, or as indicated, and antagonists were tested in the presence of 100 µM serotonin.

### Other methods

Protein concentration was determined by the Lowry or Bradford protein assay, using commercial kits from BioRad. Indirect ELISA was performed according to standard methods in 96-well plates coated with Sm5HTR peptides (1 µg/well) and serial dilutions (1∶100,000-1∶500) of rabbit Sm5HTR antiserum, pre-immune or peptide purified Sm5HTR antiserum followed by HRP-labeled goat anti-rabbit IgG at 1∶2000 dilutions. Statistical analyses of receptor activity were done using the Prism software package for Macintosh (v3.0a, Graphpad Software, San Diego, CA, USA). ClustalW protein sequence alignments and the construction of a phylogenetic tree were performed using MacVector 7.1.1 (Accelrys, Inc). Trees were built according to Neighbour-Joining method using the Best Tree mode available in MacVector and were verified by bootstrap analysis with 1000 replicates.

## Results

### Cloning and sequence analysis of Sm5HTR

**Figure 1:**
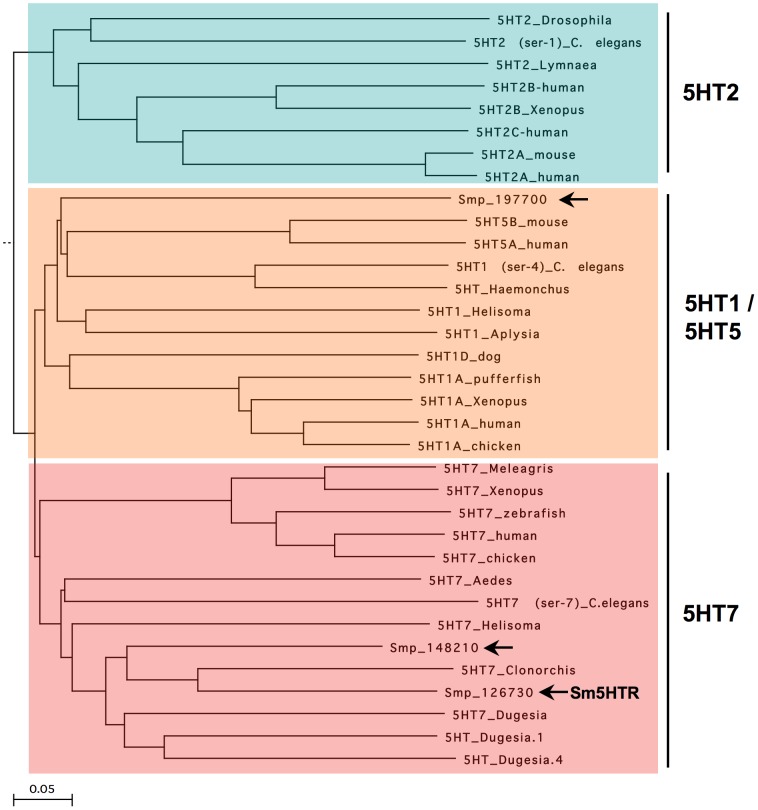
Phylogenetic tree of serotonin receptors. The amino acid sequences of known and predicted serotonin receptors were aligned using the ClustalW method and a Neighbor-Joining Best Tree was constructed from the alignment. The alignment included representative examples of all major classes of serotonergic GPCRs but only three clades are shown for simplicity. These include type 2 serotonin receptors (5HT2), type 7 (5HT7) and structurally related type 1 and type 5 receptors (5HT1/5). The outgroup in the alignment (not shown) was a distantly related rat metabotropic glutamate receptor (NP_058707). The tree was rooted to the outgroup and was tested by bootstrap analysis with 1000 iterations. The length of the branches is proportional to the genetic distance between sequences. Sequences were all obtained from the National Center for Biotechnology Information (NCBI). *S. mansoni* sequences are designated by their *S. mansoni* GeneDB “smp”designation number and are marked by arrows. Accession numbers for the remaining sequences are as follows: 5HT2_Drosophila (NP_730859); 5HT2_Lymnae (AAC16969.1), 5HT2(ser-1)_C. elegans (AAC15778); 5HT2B_Xenopus (NP_001082744); 5HT2A_human (NP_000612); 5HT2A_mouse (NP_766400); 5HT2B_human (NP_000858); 5HT2C_human (NP_000859); 5HT5B_mouse (NP_034613.2); 5HT5A_human (NP_076917.1); 5HT1_Helisoma (AAQ95277.1); 5HT1A_Xenopus (NP_001079299.1); 5HT1A_human (NP_000515.2); 5HT1A_chicken (NP_001163999.1); 5HT1D_dog (P11614); 5HT1A_pufferfish (O42385); 5HT1_Aplysia (NP_001191550); 5HT1(ser-4)_C. elegans (NP_497452); 5HT_Haemonchus (AAO45883); 5HT7_Xenopus (NP_001079253.1); 5HT7_human (NP_062873.1); 5HT7_chicken (NP_001167605.1) 5HT7_Helisoma (AAQ84306.1); 5HT7_Meleagris (XP_003206102); 5HT7_zebrafish (XP_690599); 5HT7_Clonorchis (GAA29051); 5HT7_Aedes (AF296125); 5HT7 (ser-7)_C. elegans (NP_741730); 5HT_Dugesia.1 (BAA22404); 5HT_Dugesia.4 (BAA22403); 5HT7_Dugesia (BAI44327.1); 5HT4_chicken (XP_414481.2); 5HT4_human (NP_000861.1); 5HT6_chicken (NP_001166911.1); 5HT6_human (NP_000862.1); 5HT6_mouse (NP_067333.1); ratmGlu.8393487; mGluR1_rat (NP_058707).

Sm5HTR was cloned by standard RT-PCR using the predicted coding sequence (Smp_126730) from the *S. mansoni* genome database [34], [35]. The resulting cDNA (NCBI Accession# KF444051) was verified by sequence analysis of three separate clones and found to be identical to the genomic prediction. The cDNA has 1393 bp and encodes a protein of 463 amino acids with a molecular weight of 52 kDa and a pI close to 10 (pI = 9.98). Sequence homology analysis places Sm5HTR within the 5HT7 clade of serotonin receptors (Fig. 1). The most similar proteins are 5HT7-like receptors from the planarian *Dugesia japonica* and a predicted genomic 5HT7-like sequence from the related bloodfluke, *Clonorchis*. Sm5HTR also shares significant homology with serotonin receptors from other invertebrate phyla, in particular molluscs, insects and, to a lesser extent, *Caenorhabditis elegans*. Besides Sm5HTR, our search of the *S. mansoni* database found at least one more predicted full-length receptor (Smp_197700) that aligns within the 5HT1-like clade (Fig. 1) and a partial 5HT7-like sequence (Smp_148210), which is lacking a transmembrane (TM) domain and is unlikely to be functional. Interestingly, no 5HT2-like receptors could be identified by BLAST analyses. There are other predicted biogenic amine receptors in the genome, some of which could prove to be serotonergic, but none that could be readily classified as 5HT2-like. 5HT2 receptors are conserved in many invertebrate phyla [29] and they are typically coupled to the inositol phospholipid/Ca^2+^ pathway [28], one of three major ancestral mechanisms of serotonin signaling. It is possible this type of receptor has been lost in the parasite, or it may be absent in the entire phylum. 5HT2-like receptors have yet to be described in flatworms, including planarians.

A detailed analysis of Sm5HTR at the protein level found all the hallmark features of family A (rhodopsin-like) GPCRs. The schistosome receptor has the characteristic 7-TM topology and all of the invariant residues that normally identify these receptors are conserved, including the DRY motif at the end of TM3 and the NPxxY motif of TM7 [40]. Also present are many of the residues that have been implicated in serotonin binding and activity. A comparison with the recently published crystal structures of two human serotonin receptors [41], [42] identified all but one of the residues of the serotonin binding pocket, including (the superscript describes the relative amino acid position according to the Ballesteros and Weinstein nomenclature [43]): D115^3.32^, C119^3.36^, T119^3.37^, Y191^5.38^, A199^5.46^, F321^6.51^, F322^6.52^, Y355^7.43^. Three of these residues, D115^3.32^, T120^3.37^ and A199^5.46^ form direct interactions with serotonin in the human receptors and are expected to be part of the core binding domain of Sm5HTR. One core binding site is missing, however; a conserved serine of TM5 that normally interacts with the indole ring of serotonin [41] is replaced with an alanine in the schistosome receptor (A195^5.42^). Although the serotonin binding pocket is for the most part conserved, the overall level of sequence homology with human receptors is relatively modest (≈40% identity) and many of the residues surrounding the core binding sites are not conserved in Sm5HTR, including residues that have implicated in drug binding to the human receptors [41], [42]. We also observed a conservative but potentially important substitution in one of the key sites for conformational activation, the canonical glutamate of the ionic-lock motif in TM6 [41], [42], which is changed to an aspartate in Sm5HTR (D300^6.30^). These differences reflect the great evolutionary distance between the schistosome and host receptors. They also highlight the potential of Sm5HTR for selective drug targeting.

### Heterologous expression studies

**Figure 2:**
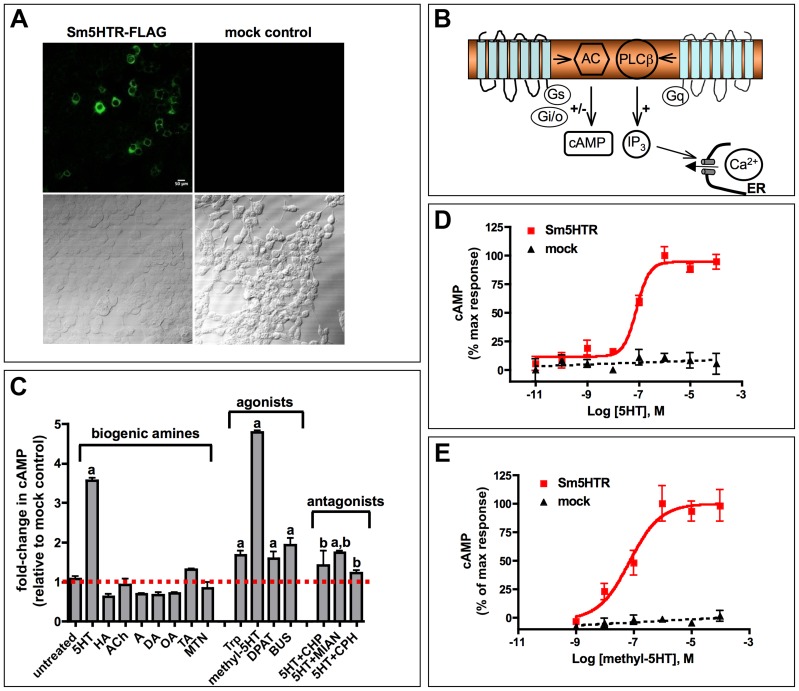
Functional expression of Sm5HTR in transfected HEK 293 cells. (A) HEK 293 cells were transfected with a plasmid expressing Sm5HTR-FLAG or empty plasmid (mock control) and probed with anti-FLAG antibody, followed by a FITC-conjugated secondary antibody. Typical *in situ* immunofluorescence results show expression of FLAG-tagged receptor in the test cells. (B) Schematic of the key signaling mechanisms of serotonin-activated GPCRs. Receptors can signal through changes in intracellular cAMP or Ca^2+^, depending on which G protein (Gs, Gi/o, Gq) is activated. Preliminary experiments showed that serotonin activation of Sm5HTR in HEK 293 cells elevated cAMP but had no effect on cytosolic Ca^2+^ (data not shown). AC, adenylate cyclase; PLCβ, phospholipase C-β; IP_3_, inositol trisphosphate; ER, endoplasmic reticulum. (C) Sm5HTR activation causes an increase in intracellular cAMP. HEK 293 cells expressing Sm5HTR were treated with biogenic amines, known serotonergic agonists and antagonists, each at 10^−4^ M. Antagonists were tested in the presence of 10^−4^ M serotonin. After incubation, the cells were lysed and the lysates were assayed for cAMP. The data were normalized relative to control cells transfected with empty plasmid (mock control). Substances tested included: serotonin (5HT), histamine (HA), acetylcholine (ACh), dopamine (DA), octopamine (OA), tyramine (TA), adrenaline (A), metanephrine (MTN), tryptamine (Trp), o-methyl-serotonin (methyl-5HT), buspirone (BUS), 8-Hydroxy-DPAT (DPAT), mianserin (MIAN), chlorpromazine (CHP), cyproheptadine (CPH). ^a^ significantly different from untreated at p<0.01; ^b^ significantly different from serotonin –induced cAMP level at p<0.01. Experiments were repeated with variable concentrations of serotonin (D) and o-methyl-serotonin (E) to obtain dose-response curves. Data were baseline-subtracted and normalized relative to the maximum agonist-induced response. Each data point is the mean and SEM of at least three independent experiments performed in duplicates or triplicates.

Sm5HTR was tested for receptor activity by expressing the protein heterologously in HEK 293 cells. In preliminary experiments, cells were transiently transfected with N-terminal FLAG-tagged Sm5HTR and probed with an anti-FLAG antibody to show that the schistosome protein could be expressed in the mammalian cell environment (Fig. 2A). Subsequent activity assays were designed based on known signaling pathways of serotonin receptors (Fig. 2B). Serotonin signaling through GPCRs is typically mediated either by cAMP, which may be increased or decreased, depending on which G protein is activated. Alternatively, serotonin can activate phospholipase C and the inositol phospholipid (IP_3_) signaling pathway, leading to an increase in cytosolic Ca^2+^. Sm5HTR was first tested for activity by measuring changes in cytosolic Ca^2+^, using a previously described fluorescence-based assay [44]. As a positive control we used a *C. elegans* serotonin receptor that is known to signal through the IP_3_/Ca^2+^ pathway [44], [45]. The results showed no effect of Sm5HTR on cytosolic calcium levels under conditions where the positive control produced a robust calcium response (data not shown). Next we tested for receptor activity using a cAMP signaling assay. Cells transfected with Sm5HTR and a control transfected with empty plasmid (mock-transfected) were treated with 100 µM test agonist or water (untreated) and then assayed for cAMP. Cells were also treated with forskolin (which increases basal cAMP) to test for the possibility of an inhibitory effect on cAMP levels. The results (Fig. 2C) show that serotonin significantly increased the level of cAMP in cells expressing the receptor compared to the mock-transfected control and the effect was inhibited by co-application of common serotonergic antagonists such as cyproheptadine or mianserin. Besides serotonin, we observed strong receptor activation in cells treated with o-methyl-serotonin and, to a lesser extent, classical (i.e. mammalian) serotonergic agonists such as tryptamine, DPAT and buspiperone. None of the other biogenic amines tested produced a significant response and therefore the receptor appears to be selective for serotonin and related substances. Experiments varying the concentration of serotonin (Fig. 2D) and o-methyl-serotonin (Fig. 2E) show that their effects are dose-dependent and have similar EC_50_ values of 61.7 nM and 72.2 nM, respectively.

### Immunolocalization studies

A polyclonal anti-Sm5HTR antibody was raised in rabbits against two specific peptides of Sm5HTR, affinity-purified and verified by ELISA against the pooled peptide antigens. Subsequent western blot analyses detected a major immunoreactive band of about 70 kDa in preparations of solubilized adult *S. mansoni* membranes (Supplemental Fig. S2). The band was shown to be specific since it was eliminated when the antibody was preadsorbed with an excess of the peptide antigens, or when it was replaced with pre-immune serum. The western positive band is larger than the expected size of Sm5HTR but this may be due to glycosylation of the receptor, a common feature among GPCRs, or the exceptionally high pI value of the protein (pI≈10.0), which could produce aberrant migration on the SDS-PAGE gel.

**Figure 3:**
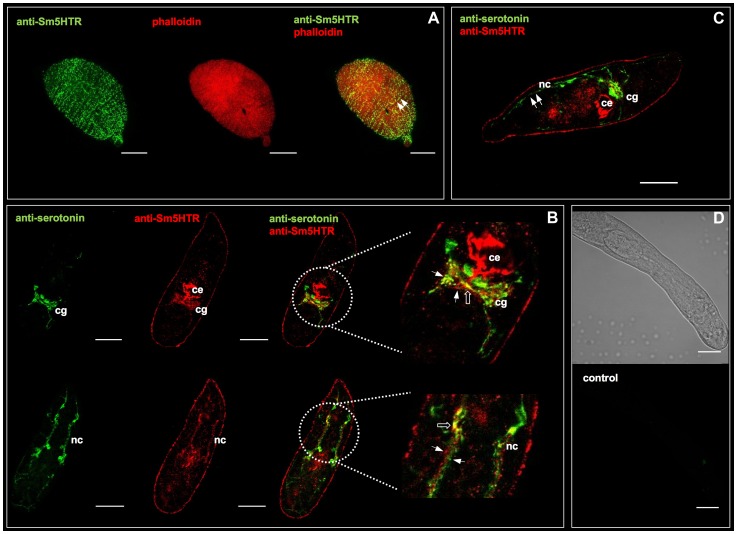
Immunolocalization of Sm5HTR in schistosomulae. *In vitro* transformed schistosomulae (3–8 days old) were probed with affinity-purified anti-Sm5HTR antibody and visualized by confocal microscopy. (A) Typical schistosomulum showing Sm5HTR (green) immunoreactivity in varicose nerve fibers along the length of the body (left panel). Animals were co-labelled with TRITC-phalloidin to visualize the musculature (middle panel) and the overlay (right panel) shows punctate bright yellow fluorescence, indicating sites of apparent co-localization between Sm5HTR and the outer circular muscles of the body wall (arrows). (B) Schistosomulae were co-labelled with anti-serotonin antibody (green) and anti-Sm5HTR antibody (red). The overlay shows close proximity between the two signals (solid arrows) in the cerebral ganglia (cg) and the main longitudinal nerve cords (nc). Sites of apparent co-localization are marked by open arrows. Sm5HTR immunoreactivity is also prominent in the developing caecum (ce). (C) Schistosomulum co-labelled with anti-serotonin antibody (green) and anti-Sm5HTR antibody (red). Lateral serotonin-containing nerve fibers (solid arrows) can be seen in close proximity to Sm5HTR immunoreactivity in the body wall region of the larva. cg, cerebral ganglia; ce, caecum; nc, nerve cord. (D) Transmission light and corresponding fluorescence image of a typical negative control. No significant fluorescence could be seen in any of the negative controls probed with secondary antibody only or antigen-preadsorbed antibody. Scale bars, 25 µm.

The tissue localization of Sm5HTR was examined first in schistosomulae by confocal microscopy using affinity-purified anti-Sm5HTR antibody. Some animals were co-labeled with a commercial antibody against serotonin to visualize the spatial organization of the receptor relative to its ligand. We also labeled the musculature in some experiments by treating the larvae with TRITC-labelled phalloidin. Schistosomulae displayed a distinctive pattern of punctate Sm5HTR immunoreactivity just beneath the surface and along the length of the body (Fig. 3A). The punctate fluorescence aligned to give the appearance of fine, varicose nerve fibers resembling beads on a string. Animals co-labeled with anti-Sm5HTR (green) and phalloidin (red) exhibited punctate bright yellow fluorescence (Fig. 3A, overlay), suggesting that the receptor is on nerve fibers that are closely associated with the musculature, or possibly on the muscle itself. Close inspection through the confocal stacks revealed that the receptor signal overlapped most closely with the outer circular muscles of the body. We tested 3- and 8-day old schistosomulae with similar results. Besides the body wall muscles, we observed significant Sm5HTR expression in the developing CNS of the larvae, including the cerebral ganglia and major nerve cords (Fig. 3B). Dual labeling with anti-Sm5HTR and anti-serotonin antibodies showed that the receptor is in close proximity to serotonergic neurons, where it can be activated by endogenously released transmitter (Fig. 3B, C). We detected apparent overlap between the two signals in discrete regions of the CNS (Fig. 3B) and therefore it is possible that some of the receptor is expressed in serotonergic neurons to control endogenous release. Outside the nervous system, we observed very strong Sm5HTR labeling in the esophagus and the developing caecum (Fig. 3B, C). The signal was observed in the wall of the gut and it could be associated with the gut epithelium or the muscle lining. We also detected receptor expression in the acetabulum and there was specific but diffuse immunoreactivity scattered throughout the parenchyma. The latter may be due to receptor expression in undifferentiated neurons, muscle or other cells that are present in the parenchyma of the immature parasite. No significant immunoreactivity was detected in controls treated with antigen-preadsorbed anti-Sm5HTR antibody or controls in which the primary antibody was omitted (Fig. 3D).

**Figure 4:**
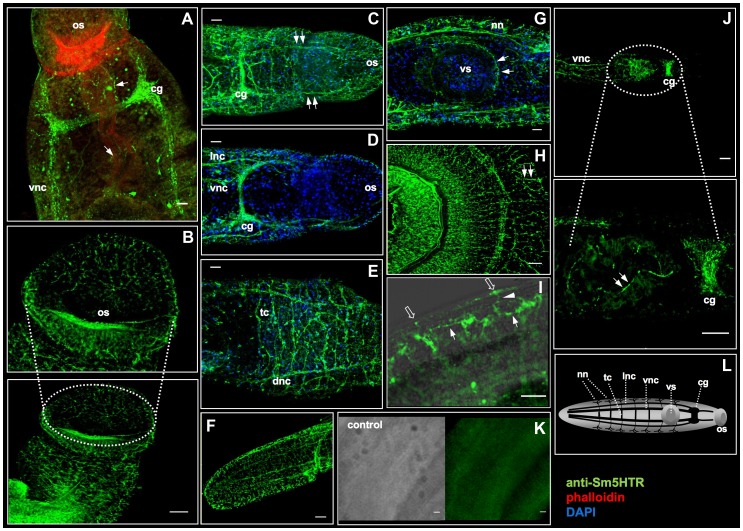
Immunolocalization of Sm5HTR in adult *S. mansoni*. Adult worms were probed with anti-Sm5HTR antibody followed by a FITC-labeled secondary antibody (green) and then examined by confocal microscopy. In some experiments, animals were co-labeled with TRITC-phalloidin (red) to visualize the musculature or DAPI (blue) to label nucleated cell bodies. (A) A typical adult male showing strong Sm5HTR green fluorescence in the cerebral ganglia (cg) and main longitudinal ventral nerve cords (vnc) of the CNS. Also visible are smaller nerve fibers innervating the oesophagus and caecum (arrows). (B) Sm5HTR immunofluorescence is apparent in anastomosing fine nerve fibers of the oral sucker (os) in a male schistosome. (C) Head region of a male schistosome showing numerous Sm5HTR-containing nerve fibers, including a pair of main nerve cords that extend anteriorly to the oral sucker (arrows). (D) Sm5HTR expression is visible both in the ventral nerve cords (vnc) and lateral longitudinal cords (lnc) in a male worm. (E) Dorsal view of the mid-body of a male worm showing high expression of Sm5HTR in the paired dorsal nerve cords (dnc) and connecting transverse commissures (tc). (F) Large numbers of Sm5HTR immunoreactive fibers in the tail region of a male worm. (G) Sm5HTR is present in the innervation of the ventral sucker (vs) (solid arrows) and the peripheral nerve net (nn) of the worm's body wall. (H) Mid-body view of a male worm showing strong Sm5HTR immuoreactivity in numerous peripheral nerve fibers extending to the worm's body wall. A higher magnification of this peripheral innervation (I) shows that Sm5HTR is present in neuronal elements of the submuscular nerve plexus (solid arrows) and also the subtegumental nerve plexus closer to the surface of the animal (open arrows). Specific immunoreactivity can be seen in apparent sensory nerve fibers (solid arrowhead) connecting the surface of the parasite to the submuscular nerve net below. (J) Sm5HTR was also detected in female schistosomes. A typical adult female shows strong immunoreactivity in the cerebral ganglia (cg) and ventral nerve cords (vnc) of the CNS, as well as smaller nerve fibers innervating the caecum and ventral sucker. No significant immunoreactivity was seen in any of the negative controls, either male worms (K) or females. (L) Diagram of key elements of the schistosome nervous system: cg, cerebral ganglia; vnc, ventral nerve cord; lnc, longitudinal lateral nerve cord; dnc, dorsal nerve cord; tc, transverse commissures; nn, nerve net; os, oral sucker; vs, ventral sucker. Scale bars, 25 µm.

Immunolocalization studies were repeated with adult worms and the results showed very high levels of Sm5HTR expression primarily in the worm's nervous system. In males, strong Sm5HTR immunoreactivity was detected in the cerebral ganglia, major longitudinal nerve cords (ventral, lateral and dorsal nerve cords) and the transverse commissures of the CNS (Fig. 4A, C–E). Labeling was visible along the entire length of the body, from the anterior end (Fig. 4A–D), to the mid-region (Fig. 4E) and the tail (Fig. 4F). Besides the CNS, we observed high levels of expression in smaller nerve fibers and plexuses of the peripheral nervous system, including the innervation of the oesophagous and caecum (Fig. 4A), oral sucker (Fig. 4B) and the acetabulum (Fig. 4G). The oral sucker, in particular, has numerous Sm5HTR-expressing nerve fibers that penetrate deep into the muscle (Fig. 4B). Strong labeling was also observed in the lateral nerve fibers and plexuses that supply the body wall muscles and the tegument in male worms (fig. 4H, I), a region that is rich in serotonergic neurons [11]. Sm5HTR immunoreactive cell bodies and small nerve fibers are clearly visible in the submuscular plexus of the worm's body wall. Some of these immunoreactive fibers can be seen extending to the surface of the worm (Fig. 4I) and are presumed to be sensory in nature. Expression of Sm5HTR in adult females was less pronounced than in the males but the labeling pattern was similar. The highest level of expression in the females was seen in the cerebral ganglia and major nerve cords of the CNS (Fig. 4J). No significant labeling could be seen in any of the negative controls tested, including peptide preadsorbed antibody controls, either in males (Fig. 4K) or females (not shown).

### Effect of Sm5HTR on larval motility

**Figure 5:**
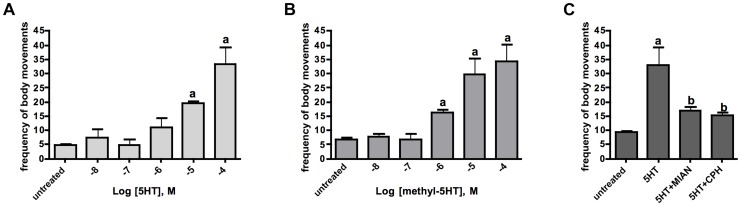
Effects of serotonin agonists and antagonists on larval motility. Schistosomulae were treated with varying amounts of serotonin (5HT) (A) or o-methyl-serotonin (B). Larval motility was quantified as the frequency of body movements (shortening and elongation) per min of observation, using an imaging assay, as described [25]. (C) Larval motility was measured in the presence of 10^−4^ M serotonin (5HT) added alone or together with a serotonin antagonist also at 10^−4^ M. Motility data are the means and SEM of 3 experiments each with 10–12 animals per treatment. ^a^ Significantly different from the untreated controls (p<0.05); ^b^Significantly different from 5HT-induced motility (p<0.05). MIAN, mianserin; CPH, cyproheptadine.

Serotonin is a known modulator of schistosome movement and therefore we questioned whether the receptor Sm5HTR might be involved in this activity. We began by testing the effects of serotonergic substances on larval motility, focusing on those substances that were shown to interact with the recombinant receptor *in vitro*. Four to six-day old schistosomulae were treated with variable concentrations of serotonin or o-methyl-serotonin in the presence and absence of antagonists, and then tested for motor activity. The results showed that serotonin and o-methyl-serotonin both stimulated larval motility in a dose-dependent manner and the effect of serotonin was virtually eliminated by co-application of either cyproheptadine or mianserin, each at 100 µM (Fig. 5). These results are consistent with the antagonist activity of the drugs towards the heterologously expressed receptor. However, cyproheptadine and mianserin could be interacting with more than one receptor and therefore additional experiments were required to verify the involvement of Sm5HTR in larval motility.

**Figure 6:**
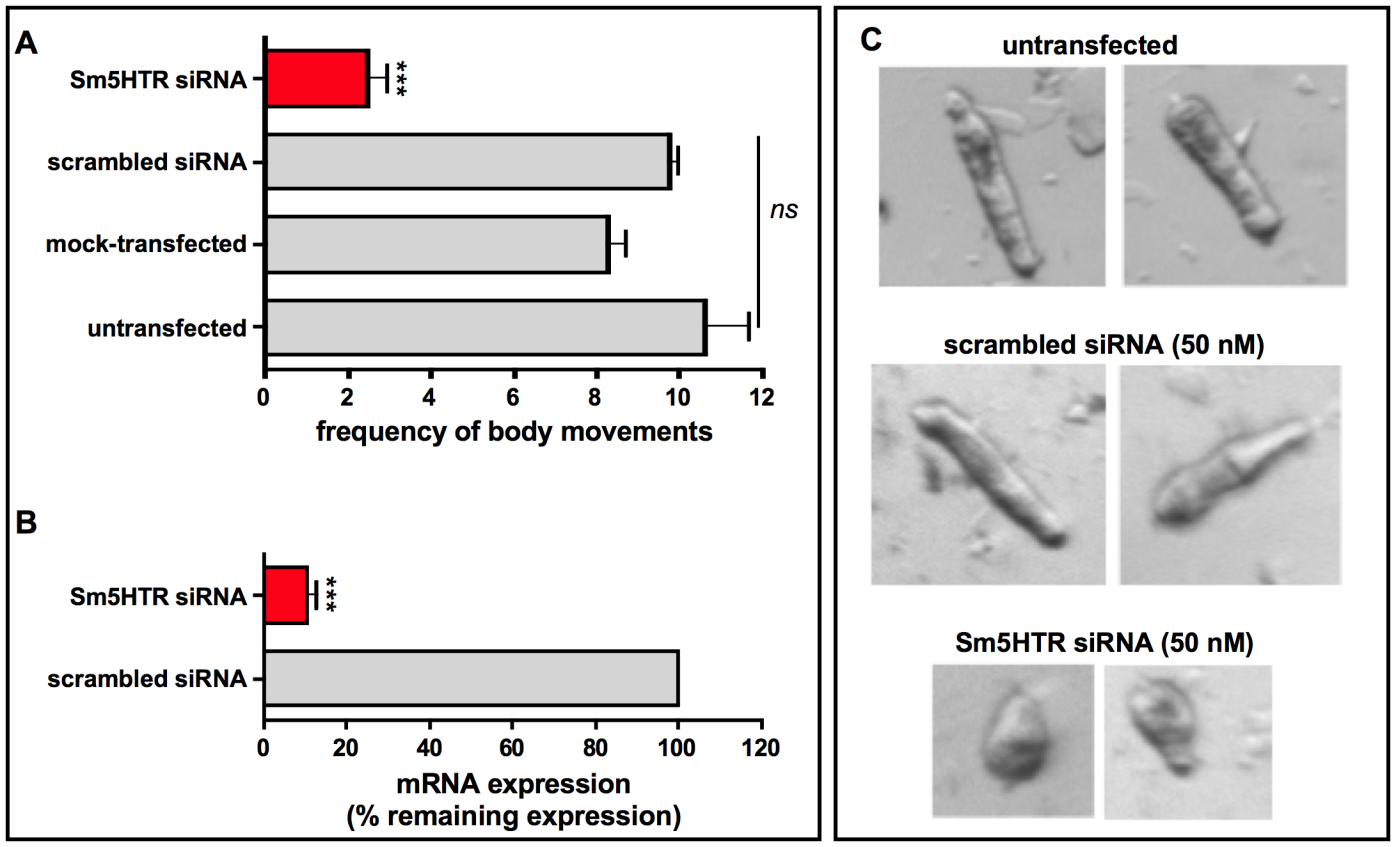
RNA interference (RNAi) in schistosomulae. Larvae were treated with 50(mock control) and cultured for 8 days. (A) Motility assays were performed using the same imaging assay described above. The data are the means and SEM of 3 experiments, each performed with at least 20 animals in duplicates. (B) For measurements of RNA expression, total RNA was isolated 8- days post-transfection and oligo-dT reverse-transcribed. Quantitative qPCR was performed with primers targeting Sm5HTR and α-tubulin as a housekeeping gene. Expression was calculated relative to the scrambled control sample, using the comparative Pfaffl method. *** Significantly different from control at p<0.0001; ns, not significant at p<0.05. (C) Representative images of schistosomulae transfected with Sm5HTR siRNA compared to scrambled siRNA or untransfected larvae. Images were obtained at 8 days post-transfection. The controls show the characteristic movement of repeated shortening and elongation, whereas the RNAi-suppressed animals are round in shape and appear unable to elongate the body.

To look more specifically at Sm5HTR, we knocked down expression of the receptor in schistosomulae by RNAi and then tested for effects on movement. Larvae were transfected with Sm5HTR-specific siRNA or an irrelevant (scrambled) siRNA as a negative control and the animals were monitored for RNAi phenotypes up to 12 days post-transfection by examination under the microscope. Previous studies have shown that RNAi treatments of 6–8 days are required to produce a measurable phenotype in larvae under these conditions [25], [36] and the same was observed in this study. All the larval RNAi data described in this study were obtained at 8 days post-transfection. To obtain representative data, we recorded video from a minimum of 20 animals per well and every experiment was repeated at least 3 times in duplicates. The results show a very pronounced hypoactive motor phenotype in the RNAi-test animals. The average motility of the larvae treated with Sm5HTR siRNA was nearly 80% less than the controls (Fig. 6A). To verify the silencing, RNA was extracted from larvae at 8 days post-transfection and receptor expression levels were measured by RT-qPCR. The results show that the decrease in movement correlates with nearly complete knockdown of Sm5HTR mRNA in the test animals compared to the scrambled siRNA (Fig. 6B). Upon closer inspection, we observed that a majority of the Sm5HTR siRNA-treated larvae were round in shape (Fig. 6C) and they lacked the normal range of body movements (shortening and elongation) that was seen in the controls. The test larvae were not completely paralyzed; we observed minor twitching of the head and tail regions but the movements were too small to be detected by our motility assay. Viability tests were performed routinely as described [36] and there was no apparent effect of test or control siRNAs on larval viability.

**Figure 7:**
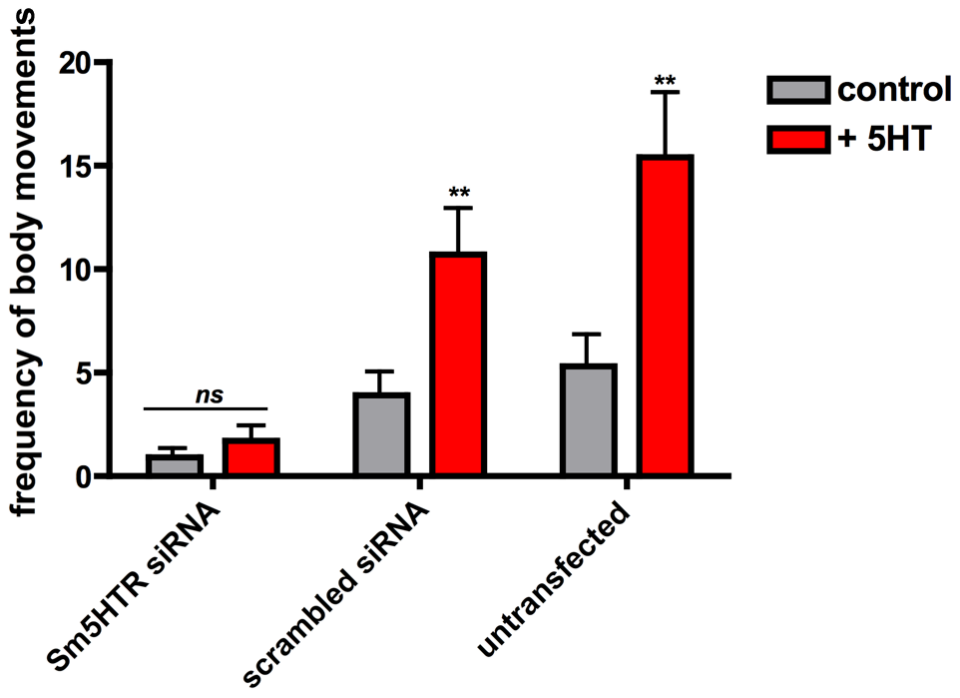
RNAi-suppressed larvae are resistant to added serotonin. RNAi was performed in cultured schistosomulae as described in Fig. 6 except that motility was measured both in the absence and presence of added serotonin (+5HT) at 10^−4^ M. Data are the means and SEM of 40 animals and 2–3 transfections per treatment. **, significantly different from basal activity (measured in the absence of serotonin) at p<0.001. ns, not significant at p<0.05.

In subsequent studies we questioned whether the RNAi-suppressed larvae were still responsive to exogenous serotonin treatment. Schistosomulae were transfected with Sm5HTR siRNA or scrambled siRNA and then tested for motor activity at day 8, as above, both in the presence and absence of 100 µM serotonin. The results show a significant effect of serotonin in the controls but not the Sm5HTR siRNA-transfected larvae (Fig. 7), indicating that the RNAi-suppressed animals are resistant to added serotonin. Based on these experiments we conclude that Sm5HTR is required for normal (endogenous) motor control in *S. mansoni* larvae and it also mediates the hyperactivity caused by exogenous application of serotonin.

### Effect of Sm5HTR on adult worm motility

To investigate the role of the receptor in adult worms it was necessary first to establish an imaging assay for quantitative measurements of movement. Measuring motility of adult worms is challenging compared to the larvae because the adults have more complex and irregular motor behaviors that are difficult to quantify. Schistosomes do not travel in culture; it is not possible to measure motility in terms of distance traveled or speed. Instead researchers typically quantify motility by measuring gross body movements, using imaging methods [46], [47]. Here we used a relatively simple imaging assay based on the publicly available software, ImageJ. Video images of adult males and females were converted to binary objects, using ImageJ, and then tracked from one frame of video to the next. A body movement was seen as a displacement of pixels between two consecutive frames, which was measured in ImageJ by subtracting one frame from the other. This way motility was calculated as the average number of subtracted pixels per frame and it was normalized relative to the total number of pixels in the binary object to account for variation in worm size (Fig. S1). In preliminary experiments, we tested the assay by treating adult males and females with substances that are known to either stimulate movement (serotonin) or inhibit movement (cyproheptadine). These experiments showed that the assay was able to quantify both an increase and decrease in movement in a manner consistent with our visual inspection of the worms (Fig. S3).

**Figure 8:**
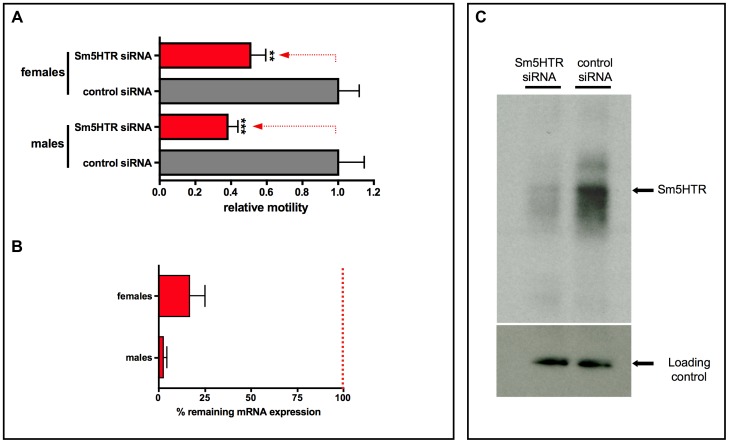
RNAi in adult worms. (A) Adult males and females were electroporated with Sm5HTR siRNA or irrelevant control siRNA and motility was recorded approximately 24 hr post-transfection, using an imaging assay. Worm movement was recorded for periods of 2 min in the presence of added serotonin (10^−4^ M) and motility data were normalized relative to the irrelevant siRNA control also treated with serotonin. Each data point is the mean and SEM of 15–19 worms obtained from three separate RNAi experiments. Motility in the RNAi – suppressed animals was significantly different from the scrambled siRNA control at p<0.001 (***) or p<0.01 (**). (B) RNAi knockdown was verified at 24 hr post-transfection by reverse transcription (RT) -qPCR analysis. The % remaining expression was determined by the Pfaffl method and was calculated relative to the corresponding male or female control siRNA group. (C) RNAi knockdown was further verified at the protein level by western blot analysis. Worms were harvested 24 hr post-transfection and membrane proteins were extracted. Equal amounts of membrane proteins from test and control worms (mixed males and females) were probed with anti-Sm5HTR antibody or an antibody against a different membrane protein (SmSERT; [25]) as a loading control. Worms electroporated with Sm5HTR siRNAs had significantly less western positive Sm5HTR protein than the control worms electroporated with irrelevant siRNA.

The same imaging assay was used to evaluate RNAi motor phenotypes in the adults. RNAi was performed using a previously described electroporation protocol [37] and the worms were monitored for changes in motor behavior after 24 hr of treatment. Experiments were extended up to 5 days and a 24–36 hr period was found to produce the strongest RNAi motor phenotype. Worm motility was measured in the presence of 100 µM serotonin to increase the level of motor activity in the worms. The results showed that adult worms treated with Sm5HTR siRNAs were significantly less motile than the controls treated with irrelevant siRNAs (Fig. 8A), both males (≈60% decrease in movement) and female worms (≈50% decrease). These results correlated with almost complete loss of Sm5HTR expression at the RNA level in males (>95% knockdown) and a significant, though less pronounced knockdown in females (≈80%), as determined by RT-qPCR (Fig. 8B). We also verified the knockdown at the protein level by western blot analysis. Solubilized membrane fractions were prepared from test and control worms (mixed males and females) and equal amounts of total membrane protein were loaded onto a gel for western blotting with affinity purified anti- Sm5HTR antibody, or an antibody targeting a different schistosome membrane protein (SmSERT [25]) as a loading control. The results show a substantial decrease in the intensity of the Sm5HTR band in the RNAi-suppressed animals compared to the controls, whereas no measurable difference was seen in the intensity of the loading marker (Fig. 8C). The qPCR and western blot analyses were both performed at the same time as the motility assays, approximately 24 hr post-transfection.

## Discussion

Serotonin has been studied in schistosomes for more than 40 years, and much has been learned about its effects in the worm, but the receptors remain elusive. Until the first annotation of the *S. mansoni* and *S. japonicum* genomes in 2009 [34], [48] most of the evidence for serotonin receptors in parasitic flatworms came from whole animal studies and analyses of crude worm extracts [see 9 for review], with relatively little biochemistry or molecular biology. The application of electrophysiology methods in the 1980s and 90s provided the first indication that serotonin receptors are present in neuromuscular structures of schistosomes [18], [19] but the proteins themselves were not identified. In recent years researchers have begun to search for receptors by mining genome and transcriptome databases, using bioinformatics tools, and a few candidate sequences have been reported [34], [35], [48], [49]. Sm5HTR is the first of these sequences to be characterized at the protein level and the first to be identified as a functional serotonin receptor. Through a comprehensive analysis involving heterologous expression studies, RNAi and immunolocalization we show that Sm5HTR is an important component of the worm's motor control system and we provide new insight into the molecular mode of action of serotonin in *S. mansoni*.

Serotonin can interact with a rich diversity of receptors, as many as seven different families and at least 13 subtypes among vertebrate and invertebrate phyla [28], [29]. The vast majority of these receptors are rhodopsin-like GPCRs and Sm5HTR is no exception. Sm5HTR shows the characteristic heptahelical topology of the GPCR superfamily and carries many of the distinctive residues of 5HTRs from other species, including residues that are directly involved in serotonin binding (e.g. D115^3.32^) and conformational activation [41], [42]. Multisequence alignments placed Sm5HTR within the 5HT7 clade, one of three major families that are common to both vertebrate and invertebrate species. 5HT7 receptors are characterized in part by their signalling mechanism, which typically involves coupling to a stimulatory Gs protein, activation of adenylate cyclase and subsequent increase in cellular cAMP [28]. Our investigation of Sm5HTR suggests the schistosome receptor has a similar mechanism of signalling. Sm5HTR was cloned by RT-PCR, expressed in HEK293 cells and then assayed for signalling activity by measuring both cAMP and Ca^2+^, the two major intracellular messengers for GPCRs. The results showed that Sm5HTR was responsive to serotonin and the mechanism was mediated by cAMP; treatment of the transfected cells with serotonin produced a dose-dependent increase in cytosolic cAMP, whereas no change in calcium was observed. Subsequent studies showed that Sm5HTR was able to recognize common serotonergic agonists and antagonists but was unresponsive to other biogenic amine transmitters or their metabolites, indicating specificity for serotonin and related substances. The mechanism of Sm5HTR is consistent with earlier studies of serotonin in schistosomes. There is ample evidence that serotonin stimulates adenylate cyclase in crude worm extracts [26], [27] and cAMP is considered to be the principal mediator of serotonin signaling in all the flatworms. The identification of Sm5HTR as a cAMP-linked receptor supports this model of activity.

Having identified Sm5HTR as a serotonin receptor *in vitro*, we began to examine its biological role in the worm. Serotonin is a powerful stimulator of motor activity in schistosomes [14]–[16]; [ see 9] and therefore we hypothesized that Sm5HTR would play an important role in motor control. To test this hypothesis we knocked down expression of the receptor by RNAi and then measured the effect on worm movement, using quantitative imaging assays. Initially the analysis was performed in schistosomulae, which are more easily transfected and generally better suited for quantitative measurements of motor phenotypes than the adults. In preliminary studies we confirmed that exogenous serotonin caused strong, dose-dependent hyperactivity in the larvae and this was inhibited by further addition of serotonin antagonists, as would be expected of a receptor-mediated mechanism. Subsequent RNAi studies showed that Sm5HTR was at least one of the receptors involved in this response. Larvae treated with Sm5HTR-specific siRNAs were clearly hypoactive compared to the controls and they were also resistant to added serotonin. RT-qPCR analyses demonstrated specific and nearly complete knockdown of Sm5HTR at the RNA level thus suggesting that the hypoactivity was due to loss of the receptor.

RNAi experiments were repeated in males and female schistosomes to assess the importance of the receptor in the adult parasites. Performing RNAi in adult worms is more challenging than the larvae, in part because of the difficulty in transfecting the worms and also the complexity of adult motor behaviours, which complicates the analysis of RNAi phenotypes. To knockdown expression of Sm5HTR we used a previously described RNAi protocol based on electroporation [37] and we also developed a simple imaging assay to quantify the complex body movements of adult males and females. These studies show that Sm5HTR is just as important for motor control in the adult worms as it is in the larvae. Animals treated with Sm5HTR-specific siRNAs were much slower than the controls and this correlated with nearly complete loss of Sm5HTR expression both at the RNA and protein levels. Interestingly the RNAi effects were seen after a relatively short treatment, only 24 hr post-electroporation. This is considerably less than the 6–8 days normally required for the detection of RNAi phenotypes in the larvae. The discrepancy could be due to differences in RNAi machinery between adults and larvae or, more likely, differences in experimental protocol. The larval and adult RNAi protocols used different transfection methods (liposome-based transfection versus electroporation) and the amounts of siRNAs used were also different (nM in the larvae compared to µM amounts in the adults), which could impact on the delivery/spreading of the siRNAs in worm tissues.

To further elucidate the mechanism of motor control, we conducted a detailed investigation of where the receptor is expressed in the worm, using confocal immunofluorescence and a specific anti-Sm5HTR antibody. A polyclonal peptide antibody was obtained for the study, affinity-purified and shown to recognize the native protein by western blot analysis. Subsequent *in situ* localization experiments revealed that Sm5HTR is abundantly expressed in the nervous system both in schistosomulae and adult worms. The receptor is present in the core elements of the CNS, the cerebral ganglia and main nerve cords, as well as the peripheral innervation of the body wall muscles and the tegument. These regions are amply innervated by serotonergic neurons [8], [11], [20] and we showed through dual labeling experiments that the receptor is expressed in close proximity to serotonin-containing nerve fibers, where it could be activated by endogenously released transmitter.

**Figure 9:**
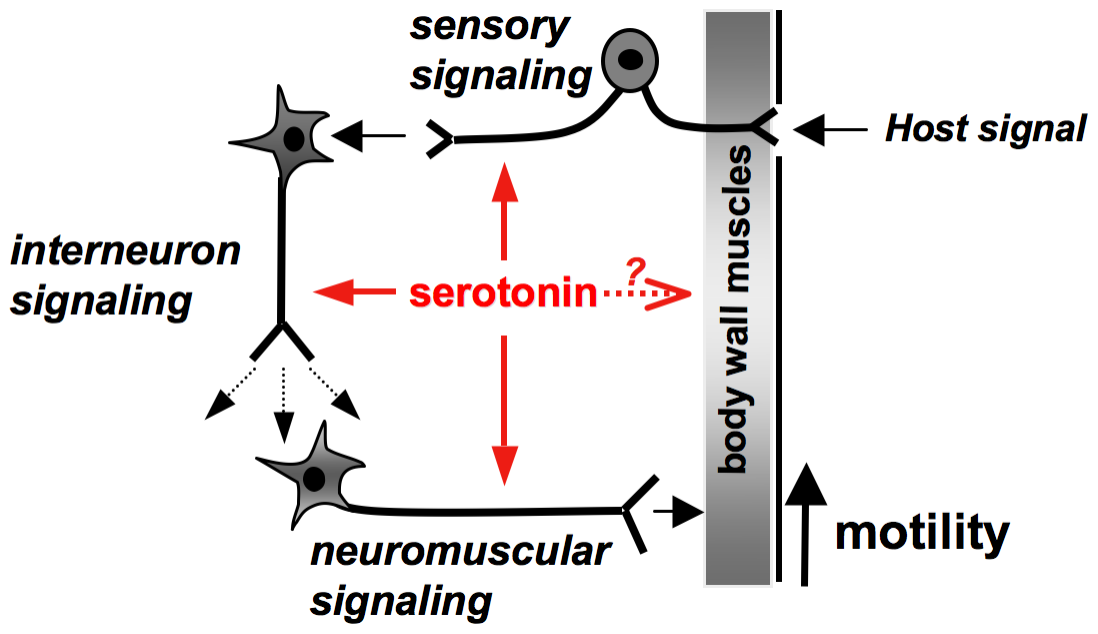
Model of serotonin signaling mediated by Sm5HTR in *S. mansoni*. Summary of mechanisms by which serotonin can stimulate worm movement. The results suggest that serotonin works through its Sm5HTR receptor to stimulate interneuronal and/or neuromuscular signaling, indirectly increasing contractility of the musculature. Serotonin may also have direct effects on the muscles but it is unclear if those effects are mediated by Sm5HTR or a different receptor. An additional mechanism by which serotonin may control movement is through modulation of sensory neuronal circuits, where Sm5HTR is abundantly expressed. These could play an important role in mediating locomotory responses to host-derived signals, for example chemotactic responses or other types of host-parasite interaction.

Combined with the RNAi studies, the expression pattern of Sm5HTR helps to explain how serotonin stimulates motor activity in the worm (Fig. 9). The fact that Sm5HTR is so prevalent in the nervous system strongly suggests that serotonin stimulates movement indirectly by modulating neuronal output to the muscles. This is also consistent with the modulatory effects of serotonin in other organisms, for example *C. elegans*, where serotonin controls locomotion indirectly by modulating the activity of particular (“decision-making”) neurons of the brain [50], [51]. The distribution of Sm5HTR suggests that serotonin is acting at more than one level in the nervous system of *S. mansoni*, targeting both “upstream” neuronal circuits of the CNS as well as “downstream” neuromuscular circuits that supply the body wall muscles (Fig. 9). One of the mechanisms by which serotonin can modulate neuronal signaling is by regulating the release of other neurotransmitters via presynaptic receptors [52]. Given that the overall effect is an increase in movement, we speculate that serotonin acts through Sm5HTR to stimulate release of myoexcitatory transmitters (e.g. neuropeptides) [see 9, 19], which in turn initiate muscle contraction. The loss of this signaling due to RNAi or drug (antagonist) inhibition of Sm5HTR could explain the hypoactive phenotypes observed in this study.

Besides neuromodulation, serotonin may have direct effects on the body wall muscles of the worm. Previous electrophysiology studies of *S. mansoni* showed that serotonin increased muscle contraction when applied either onto preparations of body wall muscle strips or dispersed muscle fibers, leading researchers to hypothesize the existence of a muscle-based receptor [18], [19]. Interestingly, these studies also found that serotonin was unable to initiate muscle contraction when added by itself, but rather potentiated the effects of other myoexcitatory signals [19], an activity consistent with the mode of action of a modulator rather than a classical neuromuscular transmitter. These effects could be mediated by neuronal Sm5HTR present in the innervation of the muscle, which would be retained in the dispersed fibers. Alternatively serotonin may interact with receptors present in the muscle fiber itself, as originally predicted. We did not see expression of Sm5HTR in the musculature of the worm but the possibility of a muscle-based receptor cannot be ruled out. It is possible that Sm5HTR is expressed in muscle but the levels are too low to be detected by our antibody, or there may be a different serotonin receptor that has yet to be identified.

The larvae were particularly disrupted by the downregulation of Sm5HTR in the RNAi experiments. Besides being much slower than the controls, the RNAi-suppressed larvae were unable to elongate their bodies to the same extent as the untreated larvae and they appeared round in shape, all indicative of major changes in the neuromuscular system. The repeated shortening and elongation of larval schistosomes is due to coordinated contraction primarily of two types of body wall muscles; contraction of the longitudinal muscles causes the body to shorten whereas the circular muscles cause cross-sectional constriction, thereby driving the elongation phase of the movement [8], [20]. Thus the rounding of the body combined with the inability to elongate in the RNAi larvae suggest that the circular muscles were particularly affected by the loss of the receptor. Serotonin is known to have different effects on different muscle types in schistosomes [18]. In *S. mansoni* sporocysts, treatment with serotonin causes both a stimulation of movement and a lengthening of the body [16], again suggesting that the circular muscles may be preferentially targeted. Our investigation of Sm5HTR is consistent with this model, in part because of the RNAi phenotype and also the distribution of the receptor in schistosomulae. Sm5HTR is highly expressed in the innervation of the body wall muscles and there were numerous sites where the receptor appeared to be in close contact with circular muscle fibers, either in nerve endings at neuromuscular junctions or possibly the muscle itself. Activation of the receptor at these sites could increase muscle contractility and ultimately stimulate movement of the larvae. However, as discussed above, Sm5HTR is also present in the brain and main nerve cords of schistosomulae and therefore there may be additional mechanisms operating at the level of the CNS that contribute to the overall effect on larval movement.

The widespread distribution of Sm5HTR both in larvae and adult worms suggests the receptor may have other activities besides motor control. Adult males, in particular, exhibited high levels of Sm5HTR in the subtegumental nerve net and sensory nerve endings of the tubercles. There is good evidence that serotonin is present in sensory neurons [11] and the distribution of these neurons is similar to that described here for the receptor. These results raise the interesting possibility that serotonin may act through Sm5HTR to modulate sensory circuits at the surface of the worm, which could represent an important mechanism of host-parasite interaction (Fig. 9). Other potential roles that emerged from this study relate to the function of the suckers and the digestive system, where the receptor is also enriched. We observed strong Sm5HTR expression in the developing caecum of larval schistosomes, suggesting a potential role for serotonin in regulating activity or development of the gut in the immature parasite. In adult worms, Sm5HTR was abundantly expressed in the innervation of both oral and ventral suckers, regions that are rich in serotonergic neurons [8], [11]. Serotonin released from these neurons could activate this receptor to control the musculature of the suckers and therefore control the worm's ability to attach to the host and feed.

Serotonin is among the oldest neurotransmitters in animal evolution and virtually all animal phyla have serotonin receptors. Even among invertebrates, most species have more than one type of serotonin receptor, not only GPCRs but also serotonin-gated ion channels. Thus we were surprised by how few serotonin receptor candidates are encoded in the *S. mansoni* genome. Although there are several sequences that show similarity to serotonergic GPCRs, only two can be readily classified based on homology, Sm5HTR, which is 5HT7-like, and the 5HT1-like receptor, Smp_197700, which has yet to be confirmed by functional analyses. A third candidate has been annotated as a putative serotonin receptor but the genomic sequence is truncated and we have not been able to clone a full length cDNA, which could suggest this is either not expressed or is a nonfunctional truncated variant. It is possible there are other, more divergent biogenic amine-like GPCRs that respond to serotonin, which would increase the repertoire of receptors available. It is also possible that some of the serotonin receptors in schistosomes are gated channels, such as the MOD1 receptor of *C. elegans* [53], which have yet to be annotated. Nevertheless, based on the available sequence information, Sm5HTR appears to be one of possibly very few serotonin receptors in schistosomes, and therefore could be responsible for most of the activities of this transmitter, not only in motor control but also others as discussed. This could explain why the receptor is so abundantly expressed, and why the RNAi targeting this single receptor produced such pronounced motor phenotypes. Sm5HTR is an important parasite receptor and a promising drug target that deserves further investigation.

## Supporting Information

Figure S1 **Schematic of adult motility imaging assay.** Key steps in the processing of video images are shown in a diagrammatic form. Further details are provided in the Methods.
(TIF)

Figure S2 **Western blot analysis of Sm5HTR.** Western blots were performed on solubilized membrane proteins obtained from adult *S. mansoni w*orms (mixed males and females). Proteins were resolved on a 4–12% gradient SDS-PAGE gel prior to immunoblotting with peptide purified anti-5HTR antibody (A) or antigen preadsorbed antibody control (B).
(TIF)

Figure S3 **Validation of adult motility assay.** Motility of adult male and female worms was quantified using the imaging described above. Basal motility was recorded first in the absence of drug treatment (untreated control). Movement was recorded again from the same worms after a 10 min treatment with 10^−4^ M serotonin (5HT) alone, or serotonin in the presence of the serotonergic antagonist, cyproheptadine (CPH), each at 10^−4^ M. The results are consistent with previously described effects of serotonin and cyproheptadine on worm motility. Motility data were normalized relative to the basal activity measured prior to drug addition and they are the means and SEM of 15–18 worms per treatment.
(TIF)
