# Supplementary figures and images for: Serotonin Signaling in Schistosoma mansoni: A Serotonin–Activated G Protein-Coupled Receptor Controls Parasite Movement
Source: PLoS Pathog. 2014 Jan 16;10(1):e1003878. doi: 10.1371/journal.ppat.1003878 (PMC3894222; doi:10.1371/journal.ppat.1003878)

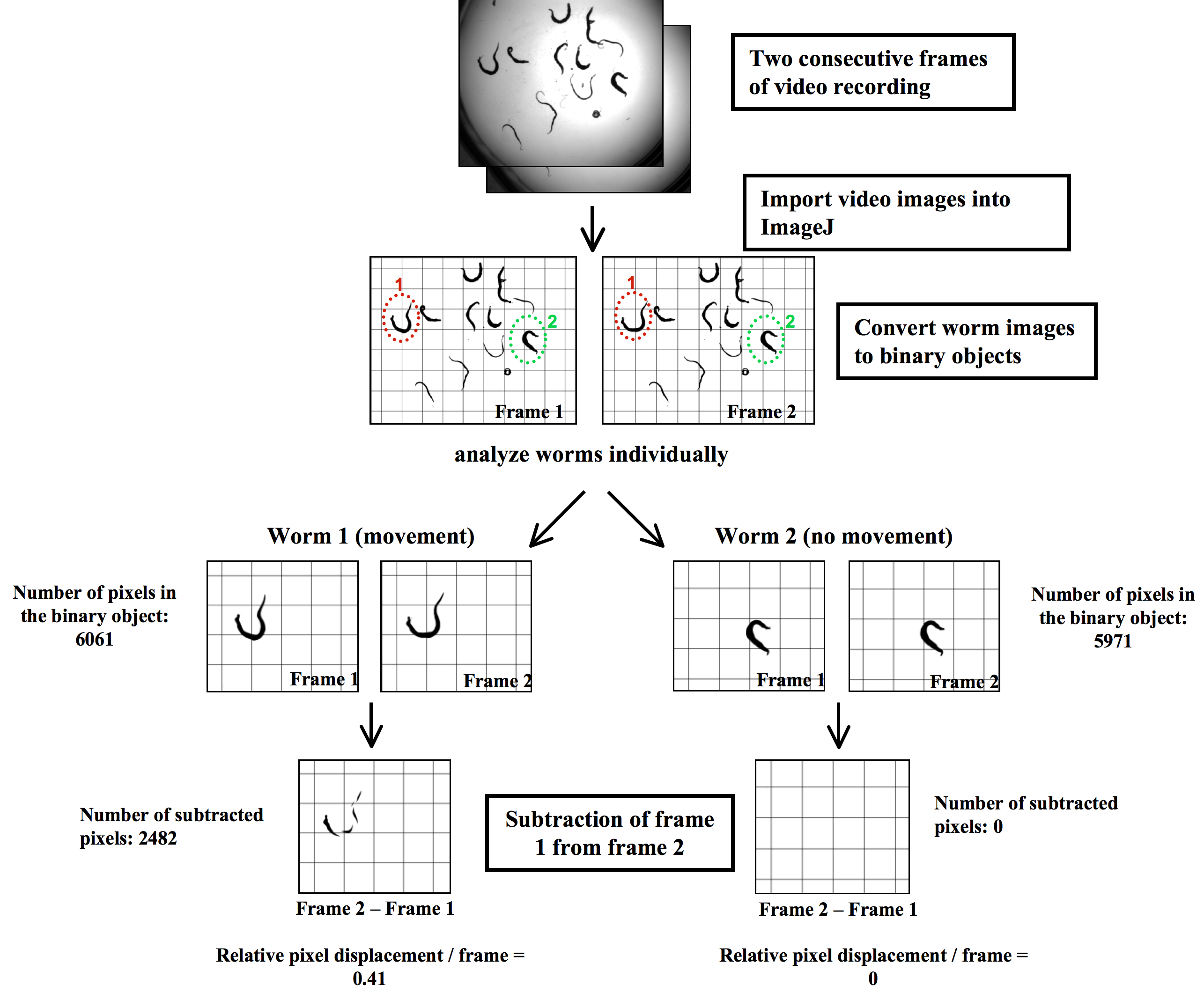

Supplement: Figure S1 — Schematic of adult motility imaging assay. Key steps in the processing of video images are shown in a diagrammatic form. Further details are provided in the Methods. (TIF) [file ppat.1003878.s001.tif]

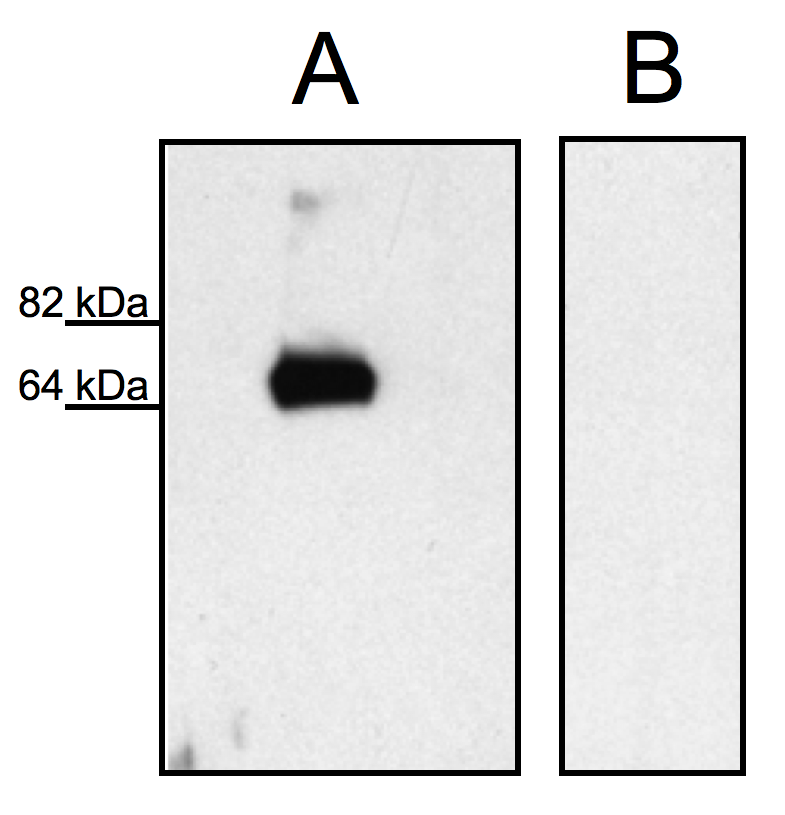

Supplement: Figure S2 — Western blot analysis of Sm5HTR. Western blots were performed on solubilized membrane proteins obtained from adult S. mansoni worms (mixed males and females). Proteins were resolved on a 4–12% gradient SDS-PAGE gel prior to immunoblotting with peptide purified anti-5HTR antibody (A) or antigen preadsorbed antibody control (B). (TIF) [file ppat.1003878.s002.tif]

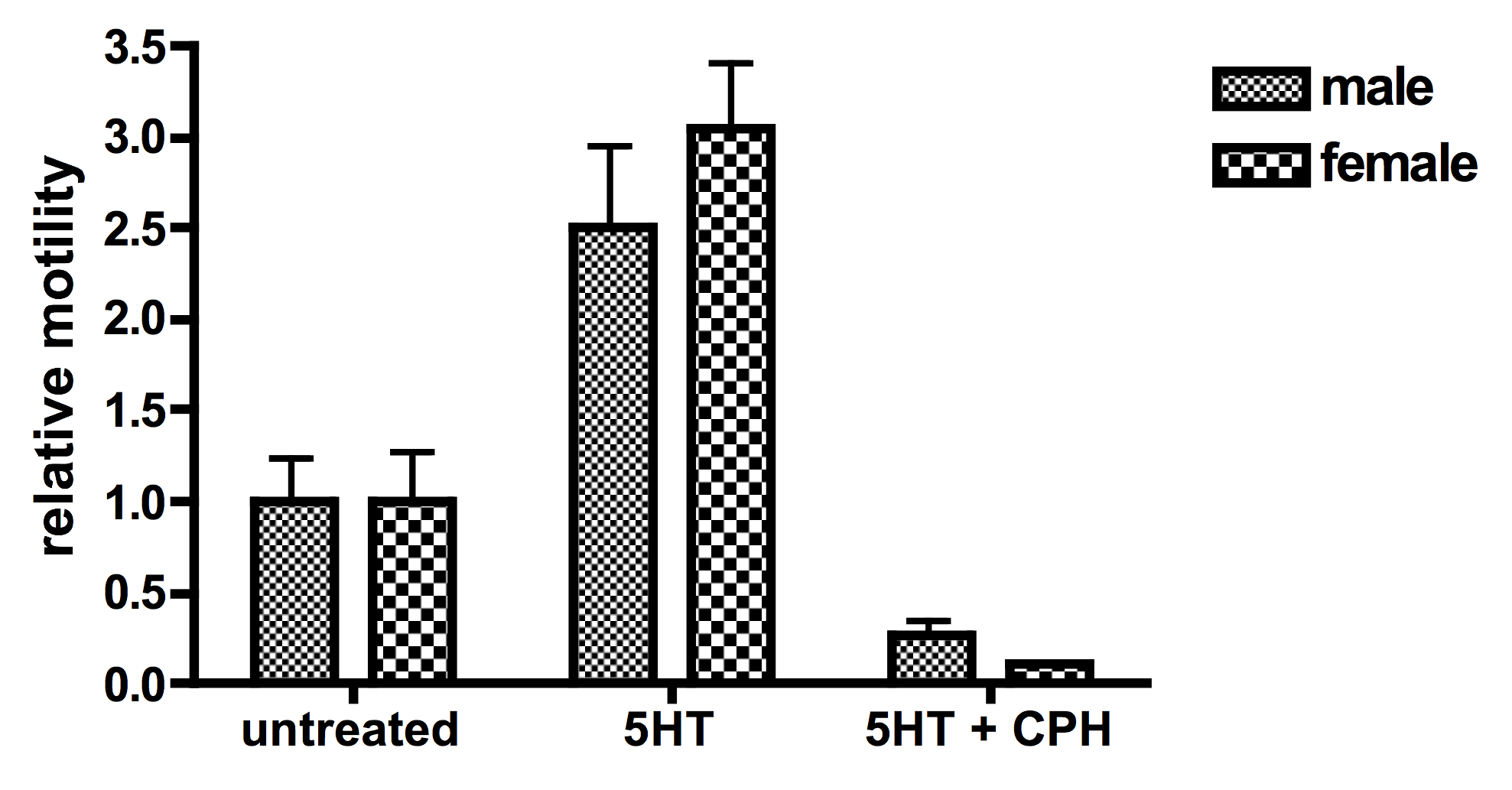

Supplement: Figure S3 — Validation of adult motility assay. Motility of adult male and female worms was quantified using the imaging described above. Basal motility was recorded first in the absence of drug treatment (untreated control). Movement was recorded again from the same worms after a 10 min treatment with 10−4 M serotonin (5HT) alone, or serotonin in the presence of the serotonergic antagonist, cyproheptadine (CPH), each at 10−4 M. The results are consistent with previously described effects of serotonin and cyproheptadine on worm motility. Motility data were normalized relative to the basal activity measured prior to drug addition and they are the means and SEM of 15–18 worms per treatment. (TIF) [file ppat.1003878.s003.tif]
